# Gonioscopy-assisted transluminal trabeculotomy versus goniotomy with Kahook dual blade in patients with uncontrolled juvenile open-angle glaucoma: a retrospective study

**DOI:** 10.1186/s12886-021-02159-z

**Published:** 2021-11-16

**Authors:** Yunsheng Qiao, Chen Tan, Xueli Chen, Xinghuai Sun, Junyi Chen

**Affiliations:** 1 Department of Ophthalmology & Visual Science, Eye & ENT Hospital, Shanghai Medical College, Fudan University, 83 Fenyang Rd, Shanghai, 200031 China; 2 State Key Laboratory of Medical Neurobiology and MOE Frontiers Center for Brain Science, Institutes of Brain Science, Fudan University, Shanghai, 200032 China; 3 NHC Key Laboratory of Myopia, Chinese Academy of Medical Sciences, and Shanghai Key Laboratory of Visual Impairment and Restoration (Fudan University), Shanghai, 200031 China

**Keywords:** Kahook dual blade, Gonioscopy-assisted transluminal trabeculotomy, Juvenile open-angle glaucoma, Minimally invasive glaucoma surgery

## Abstract

**Background:**

To compare the efficacy and safety of gonioscopy-assisted transluminal trabeculotomy (GATT) and Kahook Dual Blade (KDB) excisional goniotomy in patients with uncontrolled juvenile open-angle glaucoma (JOAG).

**Methods:**

Thirty-three patients (46 eyes) were included in this single-center, retrospective, comparative study and treated with GATT (36 eyes) or KDB goniotomy (13 eyes). Intraocular pressure (IOP), number of glaucoma medications, adverse events, and additional anti-glaucoma procedures were collected during pre- and postoperative visits. Surgical success was defined as 6 mmHg ≤ IOP ≤ 18 mmHg and ≥ 20% IOP reduction from baseline with (partial success) or without (complete success) IOP-lowering medications.

**Results:**

The mean ± SD preoperative IOP was 30.48 ± 12.9 mmHg and 26.08 ± 13.1 mmHg (*P* = 0.164) on 3.71 ± 0.46 and 3.08 ± 0.86 (*P* = 0.023) glaucoma medications in GATT and KDB group, respectively. At 3 months, the mean ± SD IOP was 15.48 ± 5.93 mmHg and 20.0 ± 10.8 mmHg after GATT and KDB, respectively (*P* = 0.072). The percentage of IOP lowering from baseline was 44.4 in the GATT group and 14.1 in the KDB group (*P* = 0.011). The mean reduction in medications was 2.6 ± 1.7 and 0.8 ± 1.2 three months after GATT and KDB, respectively (*P* < 0.001). Cumulative proportion of partial and complete success were 65.6 and 44.7% in the GATT group, 30.8 and 15.4% in the KDB group at 6 months. Additional procedures were required in 13.9% of cases after GATT and in 61.5% after KDB (*P* = 0.001). Patients in the GATT group with prior anti-glaucoma procedures and postoperative IOP spikes were more likely to fail, while those with complete trabeculotomy had a better prognosis.

**Conclusions:**

Reduction of IOP and medications were greater after GATT in uncontrolled JOAG eyes. Whereas, more additional IOP-lowering procedures were required after KDB goniotomy.

**Trial registration:**

This study was registered under the Chinese Clinical Trial Registry (ChiCTR2000034172, 27/06/2020).

**Supplementary Information:**

The online version contains supplementary material available at 10.1186/s12886-021-02159-z.

## Background

Juvenile open-angle glaucoma (JOAG) is considered to be a subtype of childhood glaucoma without ocular enlargement, the latest age of onset may extend to 30 or 40 years [1]. The tendency to rapid progression and resistance to medical treatment distinct JOAG from adult-onset primary open-angle glaucoma (POAG) [2]. Thus, surgeries are often indicated. Both traditional filtration procedures and drainage implants were reported to be effective in treating JOAG. However, high rate of postoperative complications cannot be disregarded [3–5].

Over the recent years, the advent of minimally invasive glaucoma surgeries (MIGS) expands the surgical options for glaucoma specialists. They are a group of ab-interno, conjunctiva-sparing procedures with moderate intraocular pressure (IOP) lowering effects and good safety profiles [6, 7]. Depending on whether aqueous humor is directed to Schlemm’s canal (SC), the supraciliary space, or subconjunctival space, MIGS can be further divided into three groups [8]. Gonioscopy-assisted transluminal trabeculotomy (GATT) is a circumferential trabeculotomy first developed by Grover et al. [9]. Using a 5–0 prolene suture or microcatheter, the trabecular meshwork (TM) is cleaved directly behind Schwalbe’s line leaving the so-called trabecular shelf [10]. On the other hand, excisional goniotomy by Kahook Dual Blade (KDB, New World Medical, Inc., Rancho Cucamonga, CA) was designed to remove a strip of TM by its parallel blades with minimal surrounding tissue damage [11]. Both angle-based MIGS aim to creating an opening in TM, thus relieving the proximal resistance for aqueous outflow.

Ultrastructural analysis of TM specimens from JOAG patients revealed that the extracellular deposits were accumulated in the cribriform layer [12]. In addition, both traditional trabeculotomy and goniotomy were shown to be effective in lowering IOP of JOAG eyes [13, 14]. Thus, it can be reasonably postulated that GATT and KDB are suitable surgical alternatives in treating JOAG. Indeed, there were, although limited, reports demonstrating the effectiveness and safety of GATT and KDB in the treatment of JOAG [15–17]. The purposes of this study were to compare the surgical outcomes between JOAG eyes receiving GATT and KDB goniotomy as a stand-alone procedure and seek to identify risk factors for surgical failure.

## Methods

This was a retrospective case series of JOAG patients who underwent either GATT with a 5–0 prolene suture or goniotomy with KDB by a single experienced glaucoma specialist (JYC) at Eye and ENT Hospital, Fudan University between August 2019 and March 2021. GATT was performed alternating with KDB goniotomy in consecutive patients. For those who required IOP lowering procedures in both eyes, the right eyes were treated with KDB goniotomy while the left eyes received GATT.

### Participants

Patients included in this study (1) were first diagnosed with JOAG between age 4 and 40; (2) had a history of elevated IOP and progressive deterioration of visual field; (3) with an open angle, and identifiable TM under gonioscopy; (4) whose target IOP was not achieved by maximal tolerable medications or/and previous surgeries. Exclusion criteria were as followed: (1) Patients with a bleeding diathesis or in anticoagulation therapy; (2) compromised visualization of angle structures due to corneal clouding; (3) history of ocular trauma or surgeries other than anti-glaucoma procedures.

### Surgical technique

#### GATT

Following a standard sterile preparation, a 23-gauge needle paracentesis track (tangentially oriented) was created in either superonasal or inferonasal quadrant which served as the entry for viscoelastic injection and thermally blunted sutures. Next, a temporal paracentesis was placed. To allow the best visualization of the nasal angle with Swan-Jacob goniolens, the patient’s head was rotated away from the surgeon for 30 degrees and the microscope was tilted accordingly. Then, a small goniotomy in the nasal TM was created with a microsurgical blade through the temporal site. 5–0 prolene sutures were used in all surgeries for catheterization and circumferential trabeculotomy. Briefly, the tip of a prolene suture was thermally blunted via electrocoagulation and inserted into SC through the goniotomy incision by microsurgical forceps introduced in the temporal site. Upon retrieving the suture tip, the TM is cleaved circumferentially by pulling both ends of the suture. Specifically, several attempts were made to ensure the maximal extent of TM was cleaved if the initial trabeculotomy was not complete (360°). These included reintroducing the suture into the SC from the original nasal incision in the opposite direction or creating an additional inferior or temporal incision if a 360° trabeculotomy was still not achieved.

#### KDB

After placing a temporal incision, the microscope and patient’s head were adjusted as described above. The KDB was then introduced via the incision and inserted into SC through TM. The device was advanced clockwise or counter-clockwise along the SC to remove TM in the nasal quadrant as much as possible. The free-floating strip of TM was removed by vitreous forceps. At the end of both procedures, the viscoelastic was removed from the anterior chamber.

### Postoperative care and follow-up

After surgery, all patients were given topical antibiotics and steroids for 3–4 weeks, which were tapered at surgeon’s discretion. Pilocarpine eyedrops were administered and gradually reduced within 3 months. Routinely, patients were followed up in the postoperative visits: 1 day, 1 week, 1 month, 3 months, and thereafter every 3 months. At each visit, the following data were documented: IOP, number of glaucoma medications, surgical complications, and requirement for additional procedures. Surgical success was defined as: (1) 6 mmHg ≤ IOP ≤ 18 mmHg, and a reduction by more than 20% from the baseline with (partial success) or without (complete success) glaucoma medications; (2) no loss of light perception; (3) no need for reoperation. The use of topical steroids and pilocarpine was not counted as glaucoma medications. Especially, transient IOP rise (≥ 30 mmHg) within 1 month postoperatively was considered as IOP spike and not classified as surgical failure. Hyphema was defined as any blood cells seen in the anterior chamber.

### Statistical methods

The clinical characteristics of studied groups were compared by Chi-square test, Mann–Whitney U test, or Student’s t-test. Longitudinal data were analyzed using paired parametric, nonparametric test, or generalized estimating equation (GEE). Kaplan-Meier methods were used to calculate cumulative rates of success. Two-stage test were introduced to compare different survival curves. Risk factors for surgical failure were identified by stepwise Cox proportional hazards regression model, stepwise logistic regression model, and GEE. Bonferroni correction was adopted in case of multiple comparisons. A *p* value less than 0.05 was considered statistically significant. All statistical analysis were performed on SPSS software (version 24.0, IBM Corp.) and visualized in R (version 3.6.1).

## Results

Data for this analysis were drawn from 46 eyes of 33 patients, 36 eyes/28 patients in the GATT group and 13 eyes/10 patients in the KDB group. Detailed demographic information were demonstrated in Table 1. There was an apparent male preponderance in JOAG patients as noted before [18]. Almost all clinical features were comparable between the two groups except that the KDB group had fewer preoperative medications (*p* = 0.023) and a longer follow-up duration (*p* = 0.002). An average of 340 ± 36.1 degrees of TM was cleaved in the GATT group, while limited range of TM was removed in the KDB group (111.9 ± 16.2 degrees) since the surgery is localized in nature.

**Table 1:** Demographic and baseline ocular characteristics

	GATT	KDB	*P* value
Number of eyes (patients)	36 (28)	13 (10)	
Age at surgery (years, mean ± SD)	27.83 ± 9.98 (*n* = 36)	23.3 ± 10.63 (*n* = 13)	0.450^a^
Sex (Male, %)	24 (85.7%)	10 (100%)	0.210^b^
History of glaucoma surgery (%)	16 (44.4%)	3 (23.1%)	0.175 ^b^
Filtration surgery	4		
KDB	3		
SLT	9	3	
Preoperative IOP (mmHg, mean ± SD)	30.48 ± 12.88 (n = 36)	26.08 ± 13.11 (*n* = 10)	0.164 ^a^
Number of preoperative medications (mean ± SD)	3.71 ± 0.46 (*n* = 35)	3.08 ± 0.86 (n = 13)	0.023^d^
Cup-to-disc ratio (mean ± SD)	0.83 ± 0.13 (n = 36)	0.88 ± 0.10 (n = 13)	0.096 ^a^
Axial length (mm, mean ± SD)	26.43 ± 1.92 (n = 36)	27.06 ± 1.52 (n = 13)	0.169^c^
CCT (μm, mean ± SD)	544.2 ± 31.01 (n = 35)	541.77 ± 28.58 (n = 13)	0.807 ^c^
RNFL (μm, mean ± SD)	64.8 ± 16.72 (n = 35)	66.0 ± 19.74 (n = 13)	0.945 ^a^
GCC (μm, mean ± SD)	62.09 ± 13.3 (n = 35)	61.58 ± 7.87 (n = 13)	0.625 ^a^
MD (dB, mean ± SD)	17.24 ± 7.77 (*n* = 34)	21.43 ± 5.25 (*n* = 11)	0.104 ^c^
Follow-up duration (days, Median, P25 ~ P75)	224.5, 129 ~ 289	415, 275 ~ 479	0.002 ^a^

*GATT* gonioscopy-assisted transluminal trabeculotomy, *KDB* Kahook dual blade, *CCT* central corneal thickness, *RNFL* retinal nerve fiber layer, *GCC* ganglion cell complex, *MD* mean deviation

^a^ Mann-Whitney U test

^b^ Chi-square test

^c^ Student’s t test

^d^ Corrected student’s t test

### Comparison of surgical efficacy

**Fig. 1:**
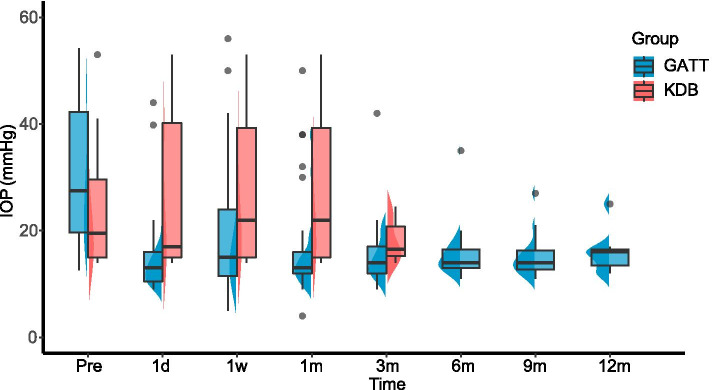
Intraocular pressure (IOP) of GATT and KDB group at pre- and postoperative visits over 12 months of follow-up. After 3 months, the majority of eyes treated with KDB goniotomy received additional IOP-lowering procedures. The gray dots represent outliers. Pre, preoperative; d, days; w, weeks; m, months

**Fig. 2:**
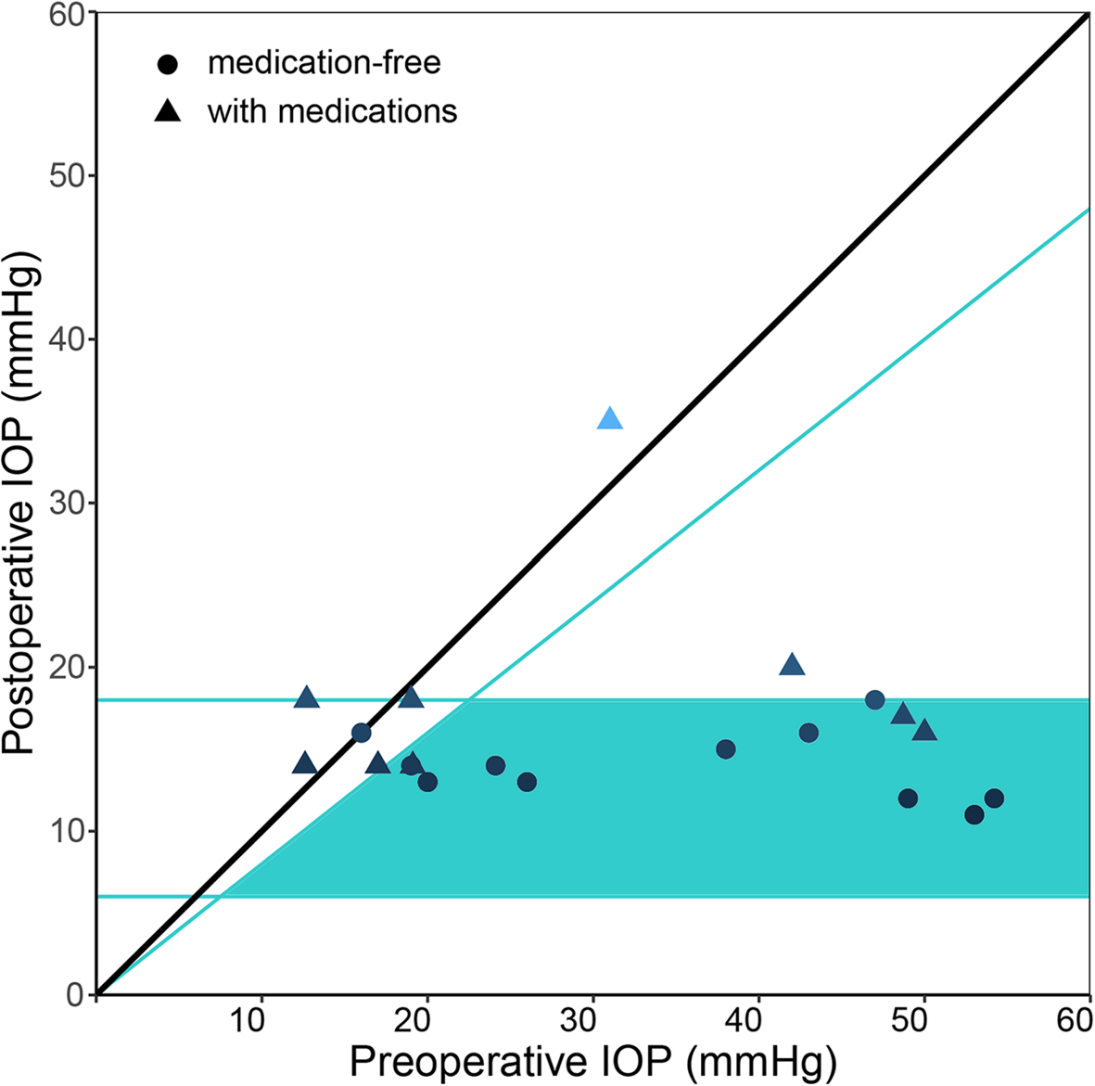
Scatterplot of postoperative intraocular pressure (IOP) at 6 months versus preoperative IOP. The diagonal line indicates no improvement. Values between horizontal cut-off lines at 18 mmHg and 6 mmHg and under the blue oblique line representing a 20% IOP reduction are considered as surgical success. Medication-free cases were visualized as blue discs

Both the IOP and number of glaucoma medications were significantly reduced at all postoperative visits compared to baseline levels for the GATT group (Table 2, Fig. 1). Average IOP decreased by 37% from 31.5 ± 14.5 mmHg preoperatively to 15.9 ± 4.9 mmHg at 6 months (*p* < 0.001). Six months after operation, 90.5% (19/21) of the eyes maintained IOP ≤ 18 mmHg, 57.1% (12/21) of the eyes achieved IOP ≤ 15 mmHg. A reduction of IOP ≥ 20% was observed in 71.4% (15/21) of the eyes with 11 medication-free cases (Fig. 2). The use of glaucoma medication decreased by 2.7 from a mean of 3.7 ± 0.5 medications preoperatively to 1.1 ± 1.4 at 6 months (*p* < 0.001). Six months after GATT, 90% (18/20) of eyes had eliminated at least one glaucoma medication. On the contrary, the IOP lowering and medication reducing effects were only short-term in the KDB group (Table 2). At 3 months postoperatively, IOP decreased by 14% from 26.1 ± 12.5 mmHg at baseline to 20 ± 10.8 mmHg (*p* = 0.1). 69.2% (9/13) of the eyes had poorly controlled IOP (> 18 mmHg) while 2 eyes were suffered from hypotony. The use of medication slightly reduced by 0.8 from 3.1 ± 0.9 medications preoperatively to 2.3 ± 1.3 at 3 months (*p* = 0.035). However, after excluding eyes complicated with hypotony, the average IOP reduction was only 3 mmHg (2.6%) on 0.5 fewer medications. Overall, both the IOP and number of medications of the GATT group descended from higher preoperative baselines to lower or comparable levels than the KDB group at all follow-up visits, although without statistical significance (Fig. 1).

**Table 2:** Intraocular pressure and medications through follow-up

	Intraocular pressure Mean (95% CI)										
Time	GATT					KDB					P for between-group comparison
	n	Preoperative	Postoperative	Reduction (%)	P for baseline comparison	n	Preoperative	Postoperative	Reduction (%)	P for baseline comparison	
1d	35	30.1 (25.9–34.3)	14.8 (12.4–17.2)	15.3 (10.1–20.6) (39)	< 0.001^a^	12	24.8 (16.7–33.0)	10.9 (8.76–13.1)	13.9 (7.36–20.5) (51)	< 0.001 ^a^	0.018^c^
1w	35	30.3 (26.0–34.5)	20.2 (16.0–24.3)	10.1 (4.87–15.3) (25)	< 0.001 ^a^	11	26.6 (17.0–36.2)	23.2 (11.2–35.2)	3.42 (−9.64–16.5) (0)	0.573 ^a^	0.877^c^
1 m	34	31.5 (27.4–35.7)	16.5 (13.3–19.8)	15.0 (9.70–20.3) (39)	< 0.001 ^a^	13	26.1 (18.2–34.0)	20.7 (11.5–29.9)	5.39 (−7.23–18.0) (4)	0.371 ^a^	0.641^c^
3 m	31	32.0 (27.5–36.5)	15.4 (13.4–17.6)	16.5 (11.8–21.2) (44)	< 0.001 ^b^	13	26.1 (18.2–34.0)	20 (13.2–26.8)	6.08 (−1.4–13.5) (14)	0.100 ^a^	0.072^c^
6 m	21	31.5 (25.3–37.7)	15.9 (13.8–17.9)	15.6 (9.04–22.2) (37)	< 0.001 ^a^	6	18.1 (13.7–22.5)	15.2 (8.13–22.2)	2.92 (−3.3–9.15) (16)	0.293 ^a^	0.499^c^
9 m	12	32.2 (24.6–39.8)	15.5 (13.1–17.9)	16.7 (10.0–23.4) (44)	< 0.001 ^a^						
12 m	7	44.2 (37.5–50.9)	16.1 (13.1–19.1)	28.0 (21.2–34.9) (62)	< 0.001 ^a^						
Medications Mean (SD)											
1w	34	3.7 (0.5)	0.9 (1.4)	2.8 (1.5)	< 0.001 ^b^	13	3.1 (0.9)	1.5 (1.8)	1.6 (1.6)	0.004 ^a^	0.259^c^
1 m	35	3.7 (0.5)	1.2 (1.5)	2.5 (1.6)	< 0.001 ^a^	13	3.1 (0.9)	1.7 (1.5)	1.4 (1.4)	0.004 ^a^	0.364^c^
3 m	32	3.7 (0.4)	1.1 (1.5)	2.6 (1.7)	< 0.001 ^b^	13	3.1 (0.9)	2.3 (1.3)	0.8 (1.2)	0.035 ^a^	0.022^c^
6 m	20	3.7 (0.5)	1.1 (1.4)	2.7 (1.5)	< 0.001 ^b^	6	3.0 (0.9)	2.3 (1.9)	0.7 (1.2)	0.235 ^a^	0.112^c^
9 m	12	3.7 (0.5)	1.1 (1.3)	2.6 (1.6)	< 0.001 ^a^						
12 m	7	3.4 (0.5)	0.9 (1.1)	2.6 (1.5)	0.006 ^a^						

*GATT* goniotomy-assisted transluminal trabeculotomy, *KDB* Kahook dual blade

^a^ Paired Student’s t test

^b^ Paired Wilcoxon signed ranks test

^c^ Mann-Whitney U test

**Fig. 3:**
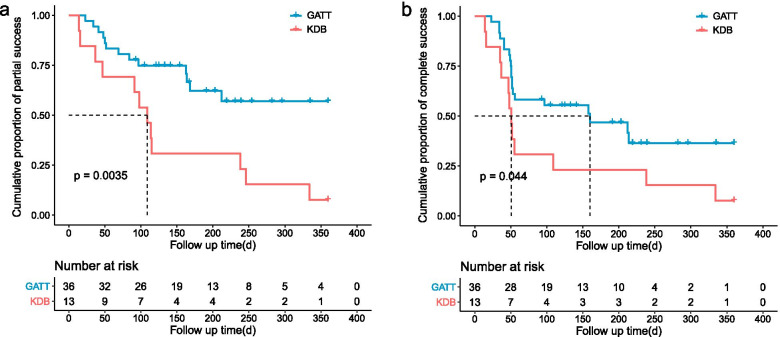
Kaplan-Meier curve demonstrating the cumulative proportions of surgical success over time separated by study group. Log-rank test indicates that the differences in survival time distributions between the GATT and the KDB group were statistically significant in terms of both partial (**a**) and complete (**b**) success. d, days

**Fig. 4:**
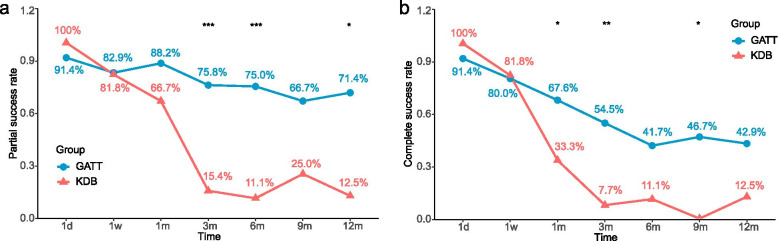
Partial (**a**) and complete (**b**) success rate at each follow-up visits. *, *p* < 0.05; **, *p* < 0.01; ***, *p* < 0.001; d, days; w, weeks; m, months

The median survival time of the GATT group was significantly longer than that of the KDB group in terms of partial (GATT: Not applicable; KDB: 109 days, *p* = 0.004, Fig. 3a) and complete success (GATT: 160 days; KDB: 51 days, *p* = 0.044, Fig. 3b). The cumulative proportions of partial and complete success were 65.6 and 44.7% for the GATT group, 30.8 and 15.4% for the KDB group at 6 months. A plunge of both partial and complete success rate for the KDB group was witnessed within 3 months after surgery, however, the success rates of two criteria remained stable around 70 and 40% respectively for the GATT group (Fig. 4). GEE model also revealed that both surgical approach and time had a significant influence on partial (*p* < 0.001 for surgical approach and time) and complete success rates (*p* = 0.002 for surgical approach and < 0.001 for time).

### Complications and secondary procedures

Summaries of postoperative complications and reoperations were presented in Table 3. Microscopic or macroscopic hyphema can be seen in all operated eyes, most of which had spontaneous resolution within one month. One eye in the GATT group with massive hyphema underwent anterior chamber washout and subsequent glaucoma valve implantation due to poor IOP control.

**Table 3:** Complications and additional IOP-lowering procedures after GATT and KDB

	GATT (N = 36)	KDB (N = 13)	*P* value
Complications			
Hyphema	36 (100%)	13 (100%)	/
Transient IOP spike	12 (33.3%)	2 (15.4%)	0.219^a^
Hypotony	1 (2.78%)	3 (23.1%)	0.022^a^
Photophobia	1 (2.78%)	/	0.735^b^
Additional procedures	5 (13.9%)	8 (61.5%)	0.001^a^
Ahmed valve	4 (11.1%)	2 (15.4%)	
SLT	2* (5.56%)	3 (23.1%)	
Excision of encapsulated bleb for previous GDD	1 (2.78%)	/	
GATT	/	3 (23.1%)	

*These two patients went through SLT and subsequent valve implantation and excision of encapsulated bleb, respectively. *IOP* intraocular pressure, *GATT* gonioscopy-assisted transluminal trabeculotomy, *KDB* Kahook dual blade, *SLT* selective laser trabeculoplasty, *GDD* glaucoma drainage device

^a^ Chi-square test

^b^ Fisher’s exact test

Transient IOP spikes were frequently encountered in the GATT group. The majority (9/12) occurred one week postoperatively. Mostly, IOP spikes were controlled with topical medications. Patients with higher preoperative IOP (OR = 12.4, *p* = 0.03) were at stake for developing IOP spikes compared to those with lower IOP baselines. Conversely, older age (OR = 0.05, *p* = 0.02) and suture dislocation to the anterior chamber or supraciliary space (OR = 0.07, p = 0.03) seemed to exert a protective effect.

Strikingly, all but two eyes in the KDB group were deemed surgical failure at the end of follow-up. Five eyes required secondary surgeries (GATT: 3 eyes; drainage implant: 2 eyes), three eyes received selective laser trabeculoplasty (SLT), and another three eyes experienced cyclodialysis cleft with hypotony. Nevertheless, maximal medications were prescribed to maintain the target IOP for the two surviving eyes. In the GATT group, 4 patients required shunt surgeries and one patient was treated with SLT and excision for encapsulated bleb of previous drainage implant.

### Identification of risk factors associated with failure in the GATT group

**Fig. 5:**
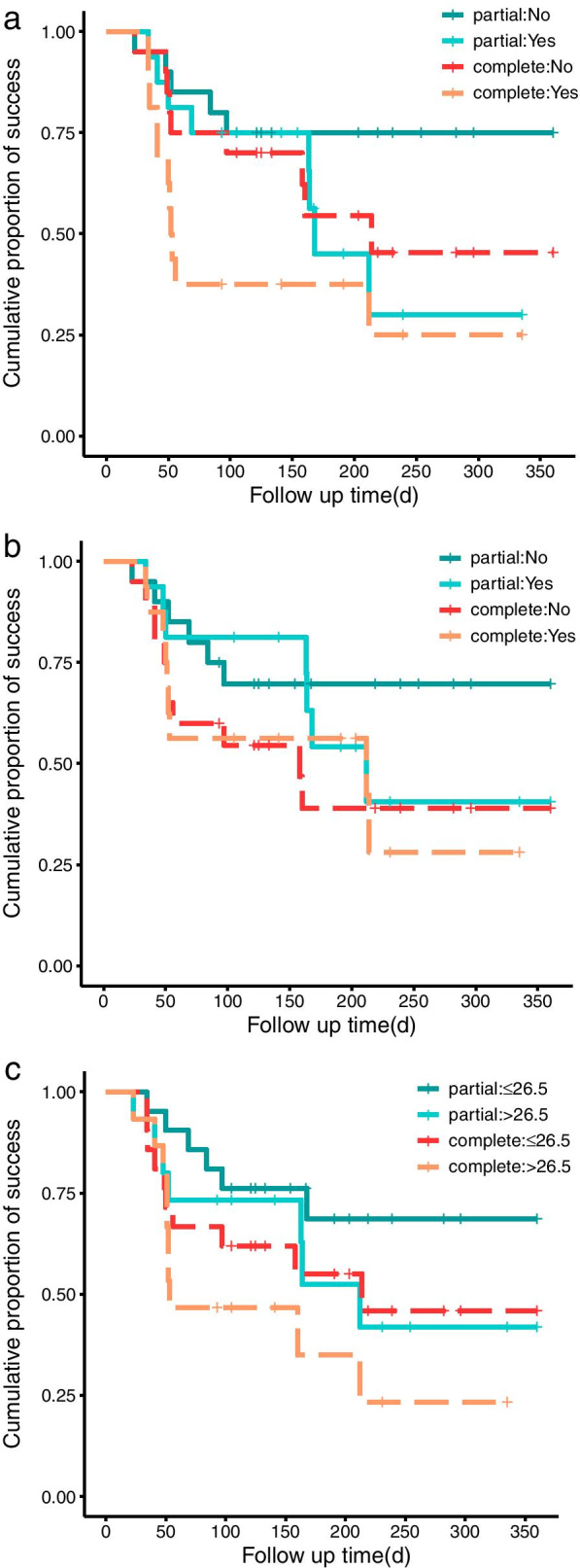
Kaplan-Meier curve of surgical success in GATT group separated by surgical history (**a**), intraoperative suture dislocation (**b**), and axial length (**c**). Partial, partial success; complete, complete success; d, days

Firstly, we categorized individuals in the GATT group according to the criteria listed in Supplementary Table 1. For partial success, the difference in the distribution of survival time between those with and without prior glaucoma surgeries was statistically significant in the long run (t_0_ = 150 days, *p* = 0.01 for both partial log-rank and Qua test, Fig. 5a). Similarly, the distribution of survival time was different for those with and without suture dislocation during operation in the late follow-up period (t_0_ = 150 days, *p* = 0.02 for both partial log-rank and Qua test, Fig. 5b). History of previous surgeries (HR = 2.84, *p* = 0.075) and the occurrence of IOP spikes (HR = 3.34, *p* = 0.05) were the major risk factors for surgical failure of GATT after applying stepwise Cox regression model. Conversely, those with complete trabeculotomy (HR = 0.21, *p* = 0.019) tend to have a better prognosis. When success rate of each postoperative visit was considered, incomplete trabeculotomy (HR = 3.89, p = 0.02) and less severe visual field damage (HR = 4.45, p = 0.02) predicted worse surgical outcomes as shown by GEE model (Supplementary Table 2).

For complete success, the distribution of survival time was significantly different for patients with and without previous glaucoma surgeries in the short term (t_0_ = 150 days, *p* = 0.03 for partial weighted Kaplan-Meier test and 0.04 for partial log-rank test, Fig. 5a), while those with shorter axial lengths (AL) enjoyed longer survival time in the long run (t_0_ = 50 days, *p* = 0.05 for partial log-rank test and 0.04 for Qua test, Fig. 5c). History of previous surgeries (HR = 2.26, *p* = 0.076) and the occurrence of IOP spikes (HR = 2.27, *p* = 0.081) were still identified as the main contributors to failure risk by stepwise Cox regression, while complete trabeculotomy was a protective factor (HR = 0.45, *p* = 0.131). In addition, lack of surgical history (HR = 0.21, *p* = 0.004) significantly reduced failure rate when incorporating other variables into GEE model (Supplementary Table 3).

## Discussions

Trabeculectomy, still considered as the gold standard in glaucoma surgery, has been reported to have a favorable IOP lowering effect at the expense of frequent occurrence of multiple complications [19–21]. The rate of surgical failure of trabeculectomy was estimated to be approximately 10% per year [22]. Because of the risks and imperfections of traditional filtration surgery as well as tube shunt implantation, there has been a major surge in innovation for angle-based procedures in the treatment of open-angle glaucoma over the past few decades [23, 24]. As two representatives, GATT and goniotomy with KDB are newly developed, ab-interno, conjunctiva-sparing, and bleb-free procedures with promising therapeutic potentials [9, 11]. The current study aimed at comparing the short-term outcomes of a series of Chinese JOAG patients who underwent GATT and KDB goniotomy.

Three months postoperatively, the mean IOP was in the mid-teens (15.4 mmHg) with a 44% reduction in the GATT group, while the average IOP fell around normal upper limit (20 mmHg) in the KDB group. After excluding eyes with cyclodialysis cleft, the percentage of IOP reduction (3%) was clinically insignificant in the KDB group. In addition, the mean IOP and number of medications in the GATT group were maintained at lower levels at all postoperative visits after initial one month compared to the KDB group. Surprisingly, high rate of postoperative complications was observed in the KDB group which led to shorter median survival times according to both partial and complete success criteria. 61.5% (8/13) of patients in the KDB group required extra IOP-lowering procedures. Among three cases underwent GATT as additional treatment, IOP control was still suboptimal in two patients.

Previous studies indicated that KDB goniotomy usually achieved final IOP in the mid-teen range regardless of baseline IOP level [25–27]. Eyes with higher preoperative IOP experienced greater reduction of IOP and glaucoma medications [28]. In well-controlled, mild-to-moderate glaucoma, however, a recent randomized controlled trial revealed that combined KDB and phacoemulsification might not be more effective than phacoemulsification alone to reach mid-teens IOP [29]. Existing evidence depicted the safe profile of KDB procedure with postoperative hyphema being the most common complication ranging from 15 to 76%, followed by IOP spikes varying from 5.7 to 18% according to different standards. Meanwhile, the rates of reoperation were relatively low (around 5%) [25–28, 30]. Up to now, no researches but one case report [17] has been described to characterize the therapeutic effect of KDB goniotomy in JOAG eyes. Thus, our findings might reflect the actual therapeutic effects of KDB treatment in this particular patient group, to which special attention should be paid.

Consistent with previous literature, GATT was found to be relatively effective and safe in our JOAG cohort. Approximately 40% of IOP lowering can be achieved with significant medication reduction in primary open-angle glaucoma [10, 31, 32]. The efficacy and safety of GATT are similar in JOAG eyes. As reported by Wang et al., the mean IOP decreased from 26.5 mmHg on 3.7 medications to 14.7 mmHg on 0.7 medications at 12 months. In one year, the qualified success rate was 81.2% with 8.5% of eyes requiring secondary procedures [15]. The IOP and medication reduction profiles of our study were comparable to that of Wang’s, although with a shorter follow-up duration. However, more eyes (13.9%) required additional procedures and the success rate (65.6%) of the treatment was relatively lower in our cohort in spite of the fact that the criteria for surgical success were similar (IOP between 6 and 18 mmHg with a reduction more than 20%). The disparities of surgical outcomes could be attributed to differences in data processing and baseline characteristics between two patient groups.

The surgical efficacy of GATT and KDB goniotomy was previously compared in primary or secondary open-angle glaucoma by Hirabayashi and colleagues [33]. They reported similar IOP lowering and medication reduction effects between KDB and GATT procedures. However, a wide variety of types of glaucoma were treated, and the baseline characteristics were not comparable to our cohort. While in pediatric glaucoma, circumferential trabeculotomy has been shown to achieve better IOP control and visual outcomes compared to goniotomy or incomplete trabeculotomy [34–36].

In this study, we identified the occurrence of IOP spikes as a risk factor for surgical failure, consistent with previous findings [15, 16, 37]. The exact mechanism of IOP spikes after GATT remained unclear, although some suggested that early spikes could be explained by retained viscoelastic materials, blood clogging, and resolution of ciliochoroidal detachment, while delayed spikes were related to steroid response. Since IOP spikes commonly occur following GATT surgery, close monitoring is essential to prevent adverse outcomes especially in younger patients with higher preoperative IOP and no suture dislocation during cannulation. Also, history of prior anti-glaucoma procedures seemed to be a risk factor for surgical failure, which was an inconsistent finding across different reports [15, 38, 39]. Although types and proportions of previous interventions varied in these studies. In addition, we found that JOAG patients benefitted from larger extent of trabeculotomy. However, opinions varied concerning the correlation between the extent of unroofed SC and IOP reduction. Some evidence supported that larger incisions contributed to a greater IOP lowering effect [16, 40], while other voices argued that complete trabeculotomy did not provide additional outflow facility [41, 42]. In practice, we noticed that the difficulty of suture cannulation differed from person to person, which presumably reflected the structural variations of SC. Failed attempts of complete trabeculotomy might indicate the defective development of aqueous drainage pathway, which potentially undermined the efficacy of GATT. Interestingly, we also observed that patients with shorter AL before surgery had a longer survival time in the long run. Although elevated IOP does not cause significant ocular expansion in JOAG eyes, fluctuation of IOP was known to induce AL changes [43]. In normal individuals, those with higher IOP tended to have longer AL [44], while AL in POAG patients was significantly longer compared to age-matched controls [45]. After filtration surgeries, AL decreased proportionally to IOP reduction [46]. Thus, we postulated that patients with longer AL could have longer disease duration or more rapid progression before treatment, which led to suboptimal surgical outcomes.

The retrospective nature of this study carries inherent limitations. For instance, it was unlikely to perform post hoc analysis of transient ciliochoroidal detachment and episcleral venous fluid wave for each patient. The small sample size limited the statistic power to perform analysis of outcomes between different subgroups. For this reason, we converted continuous variables (eg. age and preoperative IOP) into dichotomous variables. In addition, limited follow-up prevented evaluation of long-term surgical outcomes. Despite the aforementioned shortcomings, the present study provided clear evidence regarding the safety and efficacy of GATT and KDB goniotomy in Chinese JOAG eyes.

In conclusion, this is the first study focusing on comparing surgical outcomes of GATT and KDB goniotomy in JOAG eyes. On the basis of our findings, GATT was preferred in medical uncontrolled surgery-naïve eyes. Future studies are needed to confirm the discrepancy in efficacy and safety between the two surgical approaches with larger sample sizes and prospective randomized designs.

## Supplementary Information

**Additional file 1: Supplementary Table 1**. Grouping criteria.

**Additional file 2: Supplementary Table 2**. Odds ratios of grouping variables computed by generalized estimating equation according to partial success criteria.

**Additional file 3: Supplementary Table 3**. Odds ratios of grouping variables computed by generalized estimating equation according to complete success criteria.

## Data Availability

The datasets used and/or analyzed during the current study are available from the corresponding author on reasonable request.

## Abbreviation

GATT: Gonioscopy-assisted transluminal trabeculotomy
KDB: Kahook dual blade
JOAG: Juvenile open-angle glaucoma
IOP: Intraocular pressure
SD: Standard deviation
POAG: Primary open-angle glaucoma
MIGS: Minimally invasive glaucoma surgeries
SC: Schlemm’s canal
TM: Trabecular meshwork
GEE: Generalized estimating equation
SLT: Selective laser trabeculoplasty
HR: Hazard ratio
CCT: Central corneal thickness
RNFL: Retinal nerve fiber layer
GCC: Ganglion cell complex
MD: Mean deviation
GDD: Glaucoma drainage device
OR: Odds ratio

## References

[1] Thau A, Lloyd M, Freedman S, Beck A, Grajewski A, Levin AV (2018). New classification system for pediatric glaucoma: implications for clinical care and a research registry. Curr Opin Ophthalmol.

[2] Turalba AV, Chen TC (2008). Clinical and genetic characteristics of primary juvenile-onset open-angle glaucoma (JOAG). Semin Ophthalmol.

[3] Tsai JC, Chang HW, Kao CN, Lai IC, Teng MC (2003). Trabeculectomy with mitomycin C versus trabeculectomy alone for juvenile primary open-angle glaucoma. Ophthalmologica.

[4] Pathania D, Senthil S, Rao HL, Mandal AK, Garudadari CS (2014). Outcomes of trabeculectomy in juvenile open angle glaucoma. Indian J Ophthalmol.

[5] Le PH, Nguyen M, Humphrey KA, Klifto MR (2021). Ahmed and Baerveldt drainage implants in the treatment of juvenile open-angle Glaucoma. J Glaucoma.

[6] Lavia C, Dallorto L, Maule M, Ceccarelli M, Fea AM (2017). Minimally-invasive glaucoma surgeries (MIGS) for open angle glaucoma: a systematic review and meta-analysis. PLoS One.

[7] Kasahara M, Shoji N (2021). Effectiveness and limitations of minimally invasive glaucoma surgery targeting Schlemm's canal. Jpn J Ophthalmol.

[8] ElMallah MK, Seibold LK, Kahook MY, Williamson BK, Singh IP, Dorairaj SK (2019). 12-month retrospective comparison of Kahook dual blade excisional Goniotomy with Istent trabecular bypass device implantation in glaucomatous eyes at the time of cataract surgery. Adv Ther.

[9] Grover DS, Godfrey DG, Smith O, Feuer WJ, Montes de Oca I, Fellman RL (2014). Gonioscopy-assisted transluminal trabeculotomy, ab interno trabeculotomy: technique report and preliminary results. Ophthalmology.

[10] Grover DS, Smith O, Fellman RL, Godfrey DG, Gupta A, Montes de Oca I, Feuer WJ (2018). Gonioscopy-assisted transluminal Trabeculotomy: An ab Interno circumferential Trabeculotomy: 24 months follow-up. J Glaucoma.

[11] Seibold LK, Soohoo JR, Ammar DA, Kahook MY (2013). Preclinical investigation of ab interno trabeculectomy using a novel dual-blade device. Am J Ophthalmol.

[12] Furuyoshi N, Furuyoshi M, Futa R, Gottanka J, Lütjen-Drecoll E (1997). Ultrastructural changes in the trabecular meshwork of juvenile glaucoma. Ophthalmologica.

[13] Kubota T, Takada Y, Inomata H (2001). Surgical outcomes of trabeculotomy combined with sinusotomy for juvenile glaucoma. Jpn J Ophthalmol.

[14] Yeung HH, Walton DS (2010). Goniotomy for juvenile open-angle glaucoma. J Glaucoma.

[15] Wang Y, Wang H, Han Y, Shi Y, Xin C, Yin P, Li M, Cao K, Wang N. Outcomes of gonioscopy-assisted transluminal trabeculotomy in juvenile-onset primary open-angle glaucoma. Eye (Lond). 2021;35(10):2848–54. 10.1038/s41433-020-01320-0 PMC8452612 33262477

[16] Chen J, Wang YE, Quan A, Grajewski A, Hodapp E, Vanner EA, Chang TC (2020). Risk factors for complications and failure after Gonioscopy-assisted transluminal Trabeculotomy in a young cohort. Ophthalmol Glaucoma.

[17] Khouri AS, Zhu Y, Sadek H (2021). Ab interno trabeculectomy with the dual blade in juvenile open-angle glaucoma. Eur J Ophthalmol.

[18] Kwun Y, Lee EJ, Han JC, Kee C (2016). Clinical characteristics of juvenile-onset open angle Glaucoma. Korean J Ophthalmol.

[19] Gedde SJ, Herndon LW, Brandt JD, Budenz DL, Feuer WJ, Schiffman JC (2012). Postoperative complications in the tube versus trabeculectomy (TVT) study during five years of follow-up. Am J Ophthalmol.

[20] Jampel HD, Musch DC, Gillespie BW, Lichter PR, Wright MM, Guire KE (2005). Perioperative complications of trabeculectomy in the collaborative initial glaucoma treatment study (CIGTS). Am J Ophthalmol.

[21] Five-year follow-up of the Fluorouracil Filtering Surgery Study (1996). The fluorouracil filtering surgery study group. Am J Ophthalmol.

[22] Minckler DS, Francis BA, Hodapp EA, Jampel HD, Lin SC, Samples JR, Smith SD, Singh K (2008). Aqueous shunts in glaucoma: a report by the American Academy of ophthalmology. Ophthalmology.

[23] Minckler DS, Baerveldt G, Alfaro MR, Francis BA (2005). Clinical results with the Trabectome for treatment of open-angle glaucoma. Ophthalmology.

[24] Craven ER, Katz LJ, Wells JM, Giamporcaro JE (2012). Cataract surgery with trabecular micro-bypass stent implantation in patients with mild-to-moderate open-angle glaucoma and cataract: two-year follow-up. J Cataract Refract Surg.

[25] Salinas L, Chaudhary A, Berdahl JP, Lazcano-Gomez GS, Williamson BK, Dorairaj SK, Seibold LK, Smith S, Aref AA, Darlington JK (2018). Goniotomy using the Kahook dual blade in severe and refractory Glaucoma: 6-month outcomes. J Glaucoma.

[26] Iwasaki K, Takamura Y, Orii Y, Arimura S, Inatani M (2020). Performances of glaucoma operations with Kahook dual blade or iStent combined with phacoemulsification in Japanese open angle glaucoma patients. Int J Ophthalmol.

[27] Hirabayashi MT, King JT, Lee D, An JA (2019). Outcome of phacoemulsification combined with excisional goniotomy using the Kahook dual blade in severe glaucoma patients at 6 months. Clin Ophthalmol.

[28] Berdahl JP, Gallardo MJ, ElMallah MK, Williamson BK, Kahook MY, Mahootchi A, Rappaport LA, Lazcano-Gomez GS, Díaz-Robles D, Dorairaj SK (2018). Six-month outcomes of Goniotomy performed with the Kahook dual blade as a stand-alone Glaucoma procedure. Adv Ther.

[29] Ventura-Abreu N, García-Feijoo J, Pazos M, Biarnés M, Morales-Fernández L, Martínez-de-la-Casa JM. Twelve-month results of ab interno trabeculectomy with Kahook Dual Blade: an interventional, randomized, controlled clinical study. Graefes Arch Clin Exp Ophthalmol. 2021;259(9):2771–81. 10.1007/s00417-021-05213-0 33907888

[30] Sieck EG, Epstein RS, Kennedy JB, SooHoo JR, Pantcheva MB, Patnaik JL, Wagner BD, Lynch AM, Kahook MY, Seibold LK (2018). Outcomes of Kahook dual blade Goniotomy with and without phacoemulsification cataract extraction. Ophthalmol Glaucoma.

[31] Aktas Z, Ucgul AY, Bektas C, Sahin KS (2019). Surgical outcomes of Prolene Gonioscopy-assisted transluminal Trabeculotomy in patients with moderate to advanced open-angle Glaucoma. J Glaucoma.

[32] Olgun A, Aktas Z, Ucgul AY (2020). XEN gel implant versus gonioscopy-assisted transluminal trabeculotomy for the treatment of open-angle glaucoma. Int Ophthalmol.

[33] Hirabayashi MT, Lee D, King JT, Thomsen S, An JA (2019). Comparison of surgical outcomes of 360° circumferential Trabeculotomy versus sectoral excisional Goniotomy with the Kahook dual blade at 6 months. Clin Ophthalmol.

[34] Lim ME, Neely DE, Wang J, Haider KM, Smith HA, Plager DA (2015). Comparison of 360-degree versus traditional trabeculotomy in pediatric glaucoma. J aapos.

[35] Mendicino ME, Lynch MG, Drack A, Beck AD, Harbin T, Pollard Z, Vela MA, Lynn MJ (2000). Long-term surgical and visual outcomes in primary congenital glaucoma: 360 degrees trabeculotomy versus goniotomy. J aapos.

[36] Celea C, Dragosloveanu S, Pop M, Celea C (2016). Comparison of 360-degree circumferential Trabeculotomy and conventional Trabeculotomy in primary pediatric Glaucoma surgery: part 1. J Pediatr Ophthalmol Strabismus.

[37] Rahmatnejad K, Pruzan NL, Amanullah S, Shaukat BA, Resende AF, Waisbourd M, Zhan T, Moster MR (2017). Surgical outcomes of Gonioscopy-assisted transluminal Trabeculotomy (GATT) in patients with open-angle Glaucoma. J Glaucoma.

[38] Cubuk MO, Ucgul AY, Unsal E (2020). Gonioscopy-assisted transluminal trabeculotomy as an option after failed trabeculectomy. Int Ophthalmol.

[39] Grover DS, Godfrey DG, Smith O, Shi W, Feuer WJ, Fellman RL (2017). Outcomes of Gonioscopy-assisted transluminal Trabeculotomy (GATT) in eyes with prior incisional Glaucoma surgery. J Glaucoma.

[40] Chin S, Nitta T, Shinmei Y, Aoyagi M, Nitta A, Ohno S, Ishida S, Yoshida K (2012). Reduction of intraocular pressure using a modified 360-degree suture trabeculotomy technique in primary and secondary open-angle glaucoma: a pilot study. J Glaucoma.

[41] Sato T, Kawaji T (2021). 12-month randomised trial of 360° and 180° Schlemm's canal incisions in suture trabeculotomy ab interno for open-angle glaucoma. Br J Ophthalmol.

[42] Manabe SI, Sawaguchi S, Hayashi K (2017). The effect of the extent of the incision in the Schlemm canal on the surgical outcomes of suture trabeculotomy for open-angle glaucoma. Jpn J Ophthalmol.

[43] Leydolt C, Findl O, Drexler W (2008). Effects of change in intraocular pressure on axial eye length and lens position. Eye (Lond).

[44] Tomlinson A, Phillips CI (1970). Applanation tension and axial length of the eyeball. Br J Ophthalmol.

[45] Pai V, Thota RS (2017). Ocular biometry in patients with primary open angle Glaucoma (POAG). J Eye Dis Disord.

[46] Francis BA, Wang M, Lei H, Du LT, Minckler DS, Green RL, Roland C (2005). Changes in axial length following trabeculectomy and glaucoma drainage device surgery. Br J Ophthalmol.

