# Supplementary material for: Gonioscopy-assisted transluminal trabeculotomy versus goniotomy with Kahook dual blade in patients with uncontrolled juvenile open-angle glaucoma: a retrospective study
Source: BMC Ophthalmol. 2021 Nov 16;21:395. doi: 10.1186/s12886-021-02159-z (PMC8594178; doi:10.1186/s12886-021-02159-z)
Supplement: Supplementary file 1 — Additional file 1: Supplementary Table 1. Grouping criteria. [file 12886_2021_2159_MOESM1_ESM.docx]

Supplementary Table 1. Grouping criteria.

| Variables | Standards |
| --- | --- |
| Age (years) | ≤ 30 = 0, > 30 = 1 |
| Baseline IOP (mmHg) | < 30 = 0, ≥ 30 = 1 |
| Previous anti-glaucoma procedures | Yes = 1, No = 0 |
| Suture dislocation during cannulation | Yes = 1, No = 0 |
| Degrees of trabeculotomy | < 330°= 0, ≥ 330°= 1 |
| Axial length (mm) | ≤ 26.5 = 0, > 26.5 = 1 |
| CCT (μm) | ≤ 556 = 0, > 556 = 1 |
| MD (dB) | ≤ 15.3 = 0, > 15.3 = 1 |
| Postoperative IOP spike | Yes = 1, No = 0 |

CCT, central corneal thickness; MD, mean deviation.
