# Supplementary material for: Gonioscopy-assisted transluminal trabeculotomy versus goniotomy with Kahook dual blade in patients with uncontrolled juvenile open-angle glaucoma: a retrospective study
Source: BMC Ophthalmol. 2021 Nov 16;21:395. doi: 10.1186/s12886-021-02159-z (PMC8594178; doi:10.1186/s12886-021-02159-z)
Supplement: Supplementary file 2 — Additional file 2: Supplementary Table 2. Odds ratios of grouping variables computed by generalized estimating equation according to partial success criteria. [file 12886_2021_2159_MOESM2_ESM.docx]

Supplementary Table 2. Odds ratios of grouping variables computed by generalized estimating equation according to partial success criteria.

| Variables (=0) | Coefficient | OR | Wald χ^2^ | P value |
| --- | --- | --- | --- | --- |
| Age | 0.560 | 1.750 | 0.480 | 0.489 |
| Baseline IOP | -0.482 | 0.618 | 0.312 | 0.576 |
| Previous anti-glaucoma surgeries | -0.839 | 0.432 | 1.818 | 0.178 |
| Suture dislocation during cannulation | 0.496 | 1.643 | 0.568 | 0.451 |
| Degrees of trabeculotomy | 1.359 | 3.892 | 5.378 | 0.020 |
| Axial length | -0.017 | 0.983 | 0.000 | 0.984 |
| CCT | 0.874 | 2.396 | 1.766 | 0.184 |
| MD | 1.494 | 4.454 | 5.272 | 0.022 |
| IOP spike | -0.987 | 0.373 | 1.283 | 0.257 |

OR, Odds ratio; IOP, intraocular pressure; CCT, central corneal thickness; MD, mean deviation.
