# Supplementary material for: Gonioscopy-assisted transluminal trabeculotomy versus goniotomy with Kahook dual blade in patients with uncontrolled juvenile open-angle glaucoma: a retrospective study
Source: BMC Ophthalmol. 2021 Nov 16;21:395. doi: 10.1186/s12886-021-02159-z (PMC8594178; doi:10.1186/s12886-021-02159-z)
Supplement: Supplementary file 3 — Additional file 3: Supplementary Table 3. Odds ratios of grouping variables computed by generalized estimating equation according to complete success criteria. [file 12886_2021_2159_MOESM3_ESM.docx]

Supplementary Table 3. Odds ratios of grouping variables computed by generalized estimating equation according to complete success criteria.

| Variables (=0) | Coefficient | OR | Wald χ^2^ | P value |
| --- | --- | --- | --- | --- |
| Age | -0.626 | 0.535 | 0.976 | 0.323 |
| Baseline IOP | 0.112 | 1.118 | 0.021 | 0.884 |
| Previous anti-glaucoma surgeries | -1.557 | 0.211 | 8.201 | 0.004 |
| Suture dislocation during cannulation | 0.281 | 1.324 | 0.112 | 0.738 |
| Degrees of trabeculotomy | 0.844 | 2.327 | 3.076 | 0.079 |
| Axial length | -0.375 | 0.687 | 0.498 | 0.480 |
| CCT | -0.226 | 0.798 | 0.125 | 0.724 |
| MD | 0.724 | 2.063 | 2.730 | 0.098 |
| IOP spike | -1.736 | 0.176 | 2.709 | 0.100 |

OR, Odds ratio; IOP, intraocular pressure; CCT, central corneal thickness; MD, mean deviation.
